# KIF11 Serves as an Independent Prognostic Factor and Therapeutic Target for Patients With Lung Adenocarcinoma

**DOI:** 10.3389/fonc.2021.670218

**Published:** 2021-04-23

**Authors:** Zhaodong Li, Bingxin Yu, Fangyuan Qi, Fan Li

**Affiliations:** ^1^ Department of Pathogenobiology, The Key Laboratory of Zoonosis, Chinese Ministry of Education, College of Basic Medicine, Jilin University, Changchun, China; ^2^ Department of Ultrasonography, The Third Hospital of Jilin University, Changchun, China; ^3^ The Key Laboratory for Bionics Engineering, Ministry of Education, China, Jilin University, Changchun, China; ^4^ Engineering Research Center for Medical Biomaterials of Jilin Province, Jilin University, Changchun, China; ^5^ Key Laboratory for Biomedical Materials of Jilin Province, Jilin University, Changchun, China; ^6^ State Key Laboratory of Pathogenesis, Prevention and Treatment of High Incidence Diseases in Central Asia, Urumqi, China

**Keywords:** *KIF11*, prognosis, therapeutic target, lung adenocarcinoma, bioinformatics

## Abstract

**Background:**

Lung adenocarcinoma (LUAD) is challenging in clinical practice due to the poor understanding of molecular mechanisms and limited therapeutic targets. Herein, the work aimed to use bioinformatics to identify a promising molecular target for LUAD therapy.

**Methods:**

Differentially expressed genes (DEGs) from the Cancer Genome Atlas (TCGA) dataset were used for a weighted gene co-expression network analysis (WGCNA) to screen the hub gene. After a prognostic estimation with meta-analysis and COX regression analysis, we performed a function analysis on the corresponding gene. The ESTIMATE and CIBERSORT methods were adopted to analyze the association of the hub gene with the tumor microenvironment (TME). A cohort of functional assays was conducted to establish the functional roles of the hub gene in A549 and PC-9 cells.

**Results:**

Our screen identified *KIF11* as a prognostic factor, which indicated the poor overall survival and the worse progression-free survival in LUAD patients. Additionally, *KIF11* was primarily involved in cell cycle, TME alteration and tumor-infiltrating immune cells proportions. *KIF11* knockdown exerted inhibitory effects on cell proliferation, migration, and invasion. Results of the flow cytometry analysis revealed that *KIF11* knockdown induced a G2/M phase arrest and improved apoptosis in LUAD cells.

**Conclusions:**

*KIF11* is essential for LUAD cell proliferation and metastasis, and it may serve as an independent prognostic factor as well as a promising therapeutic target for LUAD patients.

## Introduction

Lung cancer is one of the most common and severe tumors in the world, leading to more than 1.4 million deaths annually (1). Lung adenocarcinoma (LUAD) is the most prevalent subtype among lung cancer patients (>40%) (2). LUAD patients with indistinct early symptoms, extensive metastasis, and chemoresistance often indicate an unfavorable overall survival (OS), and the 5-year survival rate of LUAD is not more than 10% (3–5). Advances in recent years, such as the identification of oncogenes and immunotherapy treatments, have provided valuable insight to guide the management of LUAD (6, 7). Tyrosine kinase inhibitors, as a molecular-targeted therapy, were reported to improve the survival of advanced-stage LUAD patients, and complement-related therapies are considered an optimum strategy for LUAD treatment (2, 8). However, in addition to the characteristics of LUAD, the limited knowledge of immune regulation mechanisms, and a lack of efficient biomarkers are major obstacles for the treatment of LUAD. There is a need to identify effective molecular targets and elucidate the potential mechanisms involved in the progression of LUAD.

**Figure 1:**
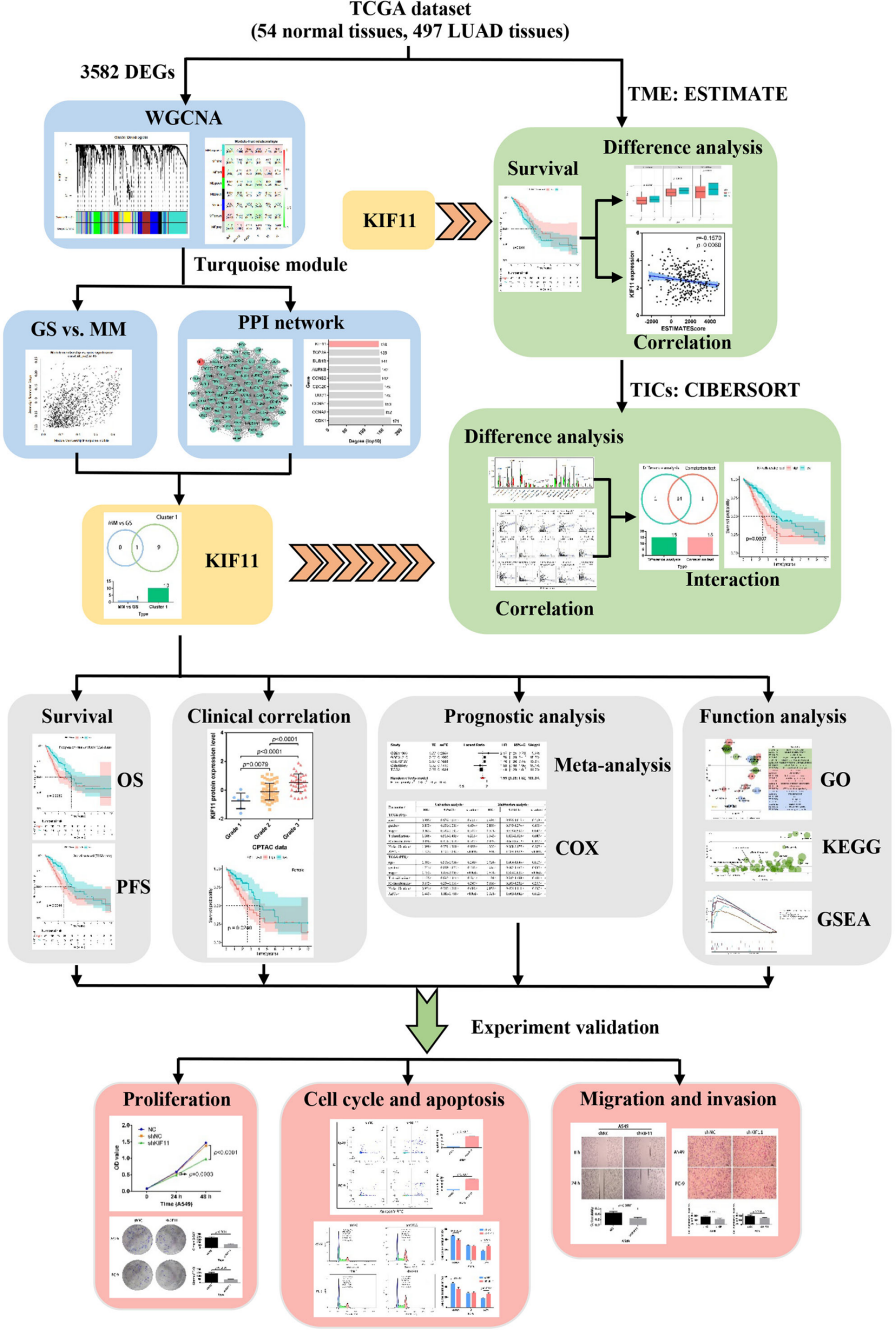
Flow diagram of the KIF11 identification workflow for LUAD. TCGA, the Cancer Genome Atlas; LUAD, lung adenocarcinoma; WGCNA, weighted gene co-expression analysis; DEGs, differentially expressed genes; OS, overall survival; PFS, progression-free survival; TME, tumor microenvironment; TICs, tumor-infiltration immune cells; GO, gene oncology; KEGG, kyoto encyclopedia of genes and genomes; GSEA, gene set enrichment analysis.

In this work, we identified a potential molecular target for LUAD treatment and described the potential mechanisms of the target in LUAD progression. We used transcriptome RNA-sequencing data (HTSeq‐FPKM) and tissue microarray data to conduct an integrated bioinformatics analysis with a series of R packages (**Figure 1**). The kinesin family member 11 (KIF11) gene was identified as a hub gene in LUAD tissues. *KIF11* belongs to the kinesin superfamily, is involved in spindle dynamics, and encodes a molecular motor protein known as Eg5, which is involved in chromosome positioning, chromosome separation, bipolar spindle construction, and driving mitosis to promote cellular proliferation (9, 10). For non-mitotic cells, Eg5 also mediates the transport of secretory proteins from the Golgi complex to the cell surface (11). Due to the essential roles of Eg5, *KIF11* has attracted interest as a promising mitotic target. Several KIF11 inhibitors have been developed including gossypol, curcumin, litrinosib, and filanesib, but have had limited success in clinical trials (10). The anticancer effects of gossypol have been demonstrated with several cancer cell types, including hepatocellular carcinoma cells, and it is currently in phase II/III clinical trials for several tumor types (10, 12). Filanesib is another promising targeted inhibitor of KIF11 that induces mitotic arrest and subsequent tumor cell death. It was reported that the combination of filanesib with dexamethasone could improve the OS to 107 months in heavily pretreated multiple myeloma patients compared with the OS of 19 months achieved with filanesib monotherapy (13). Although it has been reported that *KIF11* is overexpressed in malignant tumors including gastric cancer, malignant mesothelioma, breast cancer, and glioblastoma (14–16), there are limited reports relevant to the function of *KIF11* in LUAD.

## Materials and Methods

### Data Collection and Screen for Differentially Expressed Genes (DEGs)

LUAD-related HTSeq‐FPKM data were download from the Cancer Genome Atlas (TCGA) database (https://cancergenome.nih.gov/). The GSE33532, GSE101929, GSE68465, GSE31210, GSE42127, and GSE11969 profiles were retrieved from the Gene Expression Omnibus (GEO) database (https://www.ncbi.nlm.nih.gov/geo/). Proteomics data regarding LUAD were extracted from the Clinical Proteomic Tumor Analysis Consortium (CPTAC) database (https://cptac-data-portal.georgetown.edu/). The clinical characteristics of the datasets are in **Supplementary Table S1**. The R package “edgeR” was used to screen DEGs from the TCGA dataset with the following parameters, an adjusted *p*-value less than 0.05 and an absolute value of the log_2_(fold change) greater than one.

### Construction of the Co-Expression Network and Protein–Protein Interaction (PPI) Network

The R package “WGCNA” was used to construct a co-expression network for DEGs with a minimum module size of thirty and a merge cut height (mergeCutHeight) of 0.25. The Pearson’s correlation between external clinical information and module eigengenes (MEs) was used to identify clinically significant modules. The gene significance (GS) and module significance (MS) were used to screen for a key module. The correlation of genes with the tumor stage (cor.geneSignificance) and the correlation of genes with MEs (cor.moduleMembership) were analyzed to identify candidate key genes. An absolute value of cor.geneSignificance greater than 0.2 and an absolute value of cor.moduleMembership greater than 0.8 were set as cutoff thresholds. The DEGs in the key module wereused for a PPI network construction. The information regarding protein interactions (a combined score of greater than or equal to 0.7) was obtained from the Search Tool for the Retrieval of Interacting Genes database (STRING) (https://string-db.org/). The PPI network was visualized using Cytoscape 3.6.0 andtheCytoscape plug-in, molecularcomplex detection (MCODE), was used tocluster modules in the PPI network with default parameters. The top tengenes with the highest degrees of connectivity in the key cluster were considered candidate hub genes. The common genes that overlapped withthe candidate hubgenes in the WGCNA analysis and PPI networkwere identified as hub genes in the study.

### Hub Gene Validation and Prognostic Significance Analysis

The TCGA dataset, GSE33532, and GSE101929 profiles were used to measure hub gene expression. A meta-analysis was conducted to verify the gene expression pattern based on Oncomine database (https://www.oncomine.org). CPTAC data and immunohistochemical images from the Human Protein Atlas (HPA) (https://www.proteinatlas.org) were used to identify the protein expression levels of the corresponding genes. The mRNA and protein expression levels of the gene were further investigated with quantitative real-time polymerase chain reaction (qRT-PCR) and western blot analysis. The R packages “survival” and “survminer” were used to perform astatistical analysis for the overall survival (OS) and progress-free survival (PFS) in LUAD patients with the Kaplan–Meier method. GraphPad Prism 7.0 was used to calculate the Pearson’s correlation among terms. The R package “meta” was used to evaluate the prognostic value of hub gene in LUAD patients. The heterogeneity among different cohorts was estimated using Cochran’s Q test and Higgin’s I^2^ statistics. Meanwhile, the R package “survival” was utilized for a Cox regression analysis.

### Function Enrichment Analysis and Tumor Microenvironment (TME) Estimation

LUAD patients were divided into high and low gene expression subgroups as determined by the median hub gene expression level. The R package “clusterProfiler” was used to perform gene ontology (GO) and a Kyoto encyclopedia of genes and genomes (KEGG) enrichment analysis. Additionally, a gene set enrichment analysis (GSEA) was used to identify the functions of the hub gene in biological processes using the KEGG and HALLMARK collections. An adjusted *p*-value less than 0.05 was considered statistically significant. The R package “ESTIMATE” was applied to estimate the communities of immune and stromal cells according to the characteristics of gene expression, and then used to obtain immune, stromal and ESTIMATE scores, which are positively associated with the proportions of immune and stromal cells, and the sum of both cell types, respectively. The CIBERSORT computational method was used to explore the relative fractions of TICs in LUAD samples.

### Cell Culture and Transfection

HBE, A549, PC-9, and NCI-H1395 cells, obtained from the Shanghai Cell Bank of Chinese Academy of Medical Sciences (Shanghai, China), were cultured in high glucose Dulbecco’s Modified Eagle’s media (DMEM; Hyclone, Logan, Utah, USA) containing 10% (v/v) fetal bovine serum (FBS; Gibco, Grand Island, NY, USA), and 1% penicillin/streptomycin (MRC, Jintan, China) at 37°C and 5% CO_2_. The sequence of short hairpin RNAs (shRNA) targeting *KIF11* (5’-TGCAGGTCAGATTTACACT-3’) was cloned into the pLKO.1 plasmid to knockdown *KIF11* expression. The scrambled sequence (5’-CCTAAGGTTAAGTCGCCCTCG-3’) was the negative control. Both pLKO.1-KIF11-shRNA (shKIF11) and pLKO.1-scramble-shRNA (shNC) were bought from the Public Protein/Plasmid Library (PPL, Nanjing, China). The X-treme GENE HP DNA Transfection Reagent (Roche, Shanghai, China) was used for cell transfection per the manufacture’s protocol.

### qRT-PCR Analysis

After extracting the total RNA with the Total RNA Extraction Kit (Solarbo, Beijing, China), reverse transcription was conducted using the first-strand cDNA synthesis kit (Invitrogen, Carlsbad, CA, USA) following the manufacturers’ protocols. The Premix Ex Taq SYBR Green PCR (TaKaRa, Dalian, China) kit was then utilized to perform RT-PCR per the manufacturer’s instructions. The primer sequences used for*KIF11*amplification are TCCCTTGGCTGGTATAATTCCA (forward) and GTTACGGGGATCATCAAACATCT (reverse). The primer sequences used for *GAPDH* amplification are GGAGCGAGATCCCTCCAAAAT (forward) and GGCTGTTGTCATACTTCTCATGG (reverse).

### Western Blot Analysis

After the isolation and quantification of total protein, proteins were separated on 6% SDS-PAGE gels and transferred onto polyvinylidene fluoride membranes (ThermoFisher, Waltham, MA, USA). Afterwards, the membranes were blocked with 5% skim milk for 2 h, incubated with primary antibodies against KIF11 (Abcam, Cambridge, UK, diluted 1:1,000, ab272220) and β-actin (Abcam, diluted 1:1,000, ab8226) at 4°C overnight, then treated with horseradish peroxidase-conjugated secondary antibody (Bioss, Beijing, China) for 1 h. The enhanced chemi-luminescence reagents (Beyotime, Shanghai, China) were used to capture images of the protein bands.

### Cell Proliferation Assays

The Cell Counting Kit (CCK)-8 kit (Beyotime) and colony formation assay were used to examine cell proliferation. Briefly, cells were seeded in 96-well plates at a density of 6,000 cells/well (a volume of 100 μl per well) and incubated overnight. The next day, the plasmids were transfected. After either 24 or 48 h, the CCK-8 solution was added to each well to evaluate the cell proliferation. For the colony formation assay, 1,000 cells per well were incubated in a 6-well plate overnight and then transfected with plasmids. Either24 or 48 h later, the medium was replaced and the cells incubated for an additional 14 days. The resulting colonies were stained with Giemsa (Beyotime) and statistically analyzed using ImageJ software (version 1.8.0).

### Wound Healing Assay

Cells were incubated in a 6-well plate overnight and transfected with plasmids. At 24 h post-transfection, the cell confluence reached 100% and the cell monolayer was scratched with a 200-µl pipette tip. Serum-free medium was added to the plates and incubated for an additional 24 h. Wound closure images were captured and used to calculate cell migration distances.

### Transwell Assays

To measure invasion, transwell membranes were enveloped with Matrigel (BD Biosciences, Erembodegem, Belgium). A total of 1 × 10^4^ transfected cells were seeded into the upper chamber with serum-free medium. The lower chamber was supplemented with 600 μl of medium supplemented with 20% FBS. The next day, the cells in the lower chamber were fixed and stained. Images of the cells were collected and statistically analyzed.

### Flow Cytometry Assays

Flow cytometry assays were used to investigate the effects of KIF11 on cell cycle progression and apoptosis. Transfected cells were collected and fixed with 70% ethyl alcohol overnight at 4 °C. The cells were next either stained with 500 μl PI/RNase staining buffer (BD Pharmingen, San Diego, CA, USA) for 15 min at 37 °C or incubated with 5 μl FITC Annexin V (BD Pharmingen), 5 μl propidium iodide (PI, BD Pharmingen) and 400 μl binding buffer for 15 min at 25 °C in the dark. The cell cycle progression and apoptosis status of each cell were analyzed on a flow cytometer (BD FACSVerse, San Jose, CA, USA).

### Statistical Analysis

Data are presented as the mean ± SD of at least three independent experiments. R software (version 3.6.0) and Graphpad prism 7.0 were used for statistical analysis. A log-rank test was used to calculate statistical differences in the Kaplan–Meier analysis. The 2^−△△C^ method was used to analyze the results of qRT-PCR. Image J (version 1.48) and Graphpad prism 7.0 was used to statistically calculate the cell mobility. Student’s t-test and one-way ANOVA were applied to assess the significant differences between groups. A p-value less than 0.05 was considered statistically significant.

## Results

### KIF11 Is a LUAD Hub Gene

**Figure 2:**
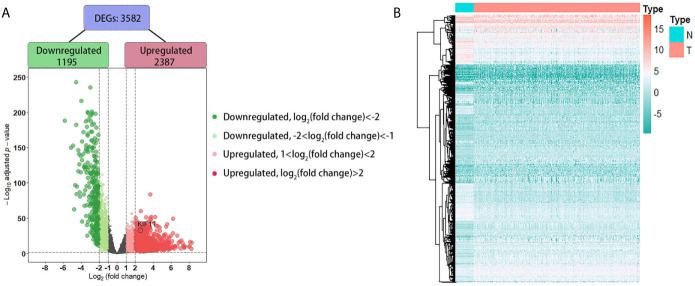
Identification of differentially expressed genes (DEGs) based on the TCGA dataset. A volcano plot depicts the 3, 582 DEGs in LUAD tissues versus normal lung samples **(A)**. An adjusted p-value <0.05 and |log2(fold change)|>1 were the cutoff criteria. A heatmap of the DEG expression profiles **(B)**. Column names indicate the sample ID; row names depict the DEG symbols.

**Figure 3:**
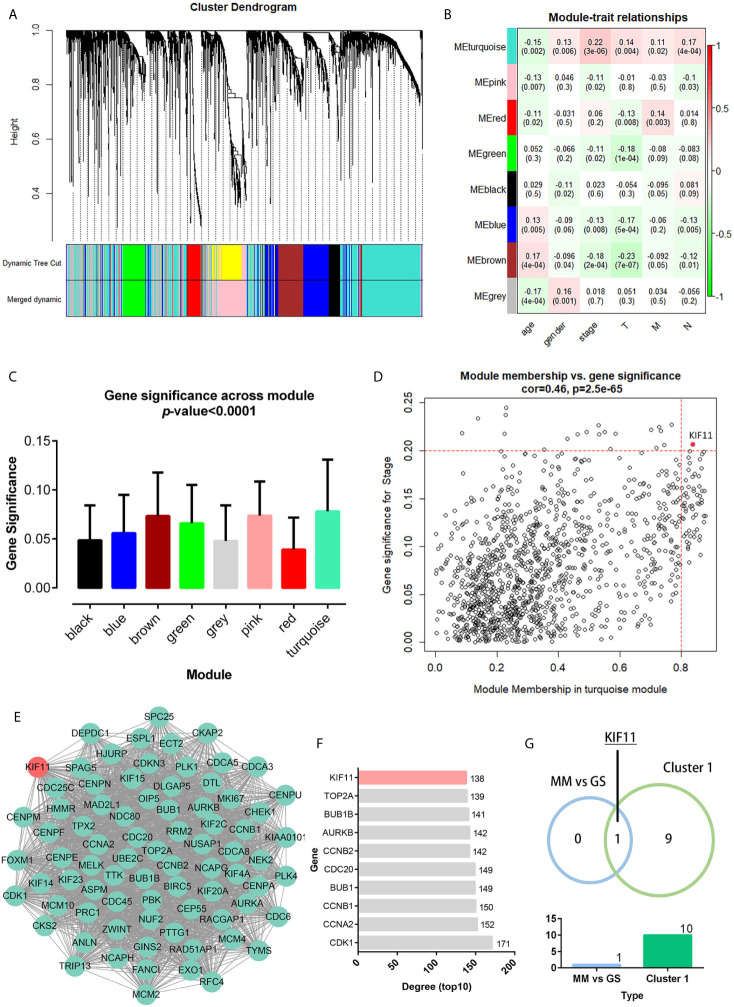
Weighted co-expression gene network and protein-protein interaction (PPI) network. Clustering dendrogram of all DEGs based on a dissimilarity measure (1-TOM) **(A)**. Heatmap of the correlation between the modules and clinical traits of LUAD **(B)**. Distribution of the average gene significance and errors in modules associated with tumor stage **(C)**. Scatter plot of the turquoise module eigengenes **(D)**. Module membership (MM) >0.8 and gene significance for stage (GS) >0.2 were set as the cutoff thresholds. The top clustering module from the PPI network of the turquoise module **(E)**. Histogram of the connectivity degrees of genes in the top cluster (top 10) **(F)**. *KIF11* was codetermined as a hub gene by the module eigengenes and connectivity degrees in a scatter plot and histogram, respectively **(G)**.

A total of 3,582 DEGs were identified in LUAD tissuescompared withnormal lung tissues, andincluded 2,387 upregulated DEGs and 1,195 downregulated DEGs (**Figure 2**). In the WGCNA for these DEGs, the power of β = 5 (scale free R^2^ = 0.87) was set as a soft-threshold to ensure a scale-free network (**Supplementary Figure S1**), and eight modules were identified based on the TCGA dataset (**Figure 3A**). These analyses indicated that the turquoise module was more related to the tumor stage than other modules (**Figure 3B**).We also found that theMS of the turquoise module was higher than those of other modules (**Figure 3C**). Herein, the turquoise module was selected as the key module and *KIF11* was identified as the candidate hub gene with the highest connectivity in turquoise module (**Figure 3D**).

Genes in the turquoise module were extracted to establish a PPI network thatincluded 634 nodes and 7,285 edges (**Supplementary Figure S2**).We identified thetop twentyclusters in the PPI network using the MCODE plug-in (**Supplementary Table S2**), which showed cluster 1 as the key cluster and hadthe highest MCODE score (68.897). We also identified the top tengeneswith the highest degrees of connectivity in the cluster 1 network (**Figures 3E, F**). A Venn diagram demonstratesthat *KIF11*is the hub gene, as codetermined by the WGCNA and PPI network (**Figure 3G**).

### KIF11 Exhibited High Expression in LUAD Samples

**Figure 4:**
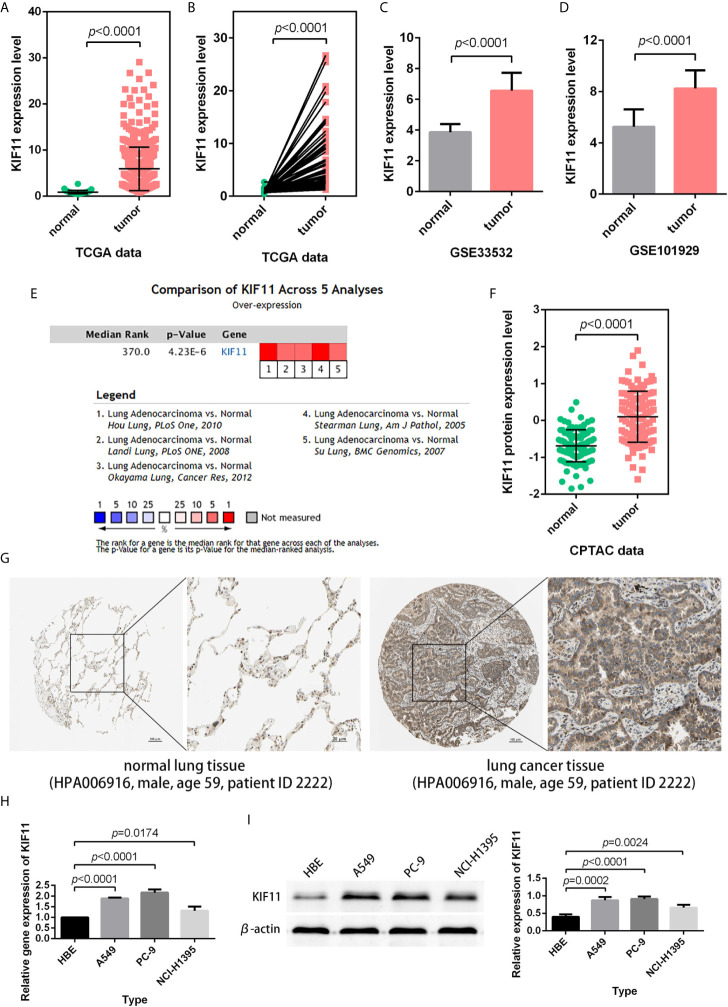
Verification of *KIF11* expression. **(A)** Unpaired and **(B)** paired difference analysis of KIF11 expression in the TCGA dataset. *KIF11* expression in the **(C)** GSE33532 and **(D)** GSE10902 profiles. KIF11 expression was upregulated among LUAD samples in **(E)** a meta-analysis, **(F)** a protein difference analysis, and **(G)** immunohistochemical images. KIF11 is highly expressed in A549, PC-9, and NCI-H1395 cells relative to HBE cells as measured by **(H)** quantitative real-time PCR and **(I)** western blot analysis.

According to the statistical analysis, *KIF11* was highly expressed in LUAD tissues compared with normal lung tissues (**Figures 4A–D**). The high expression of *KIF11*in LUAD samples was also validated in a meta-analysis containing five cohorts (Hou Lung, Landi Lung, Okayama Lung, Stearman Lung, and Su Lung, **Figure 4E**) (17–21). In addition, both CPTAC data and immunohistochemical images from HPA indicated high levels of KIF11 protein in LUAD tissues (**Figures 4F, G**). We also found the mRNA and protein expression levels of *KIF11* were distinctively upregulated in A549, PC-9, and NCI-H1395 cells versus those in HBE cells (**Figures 4H, I**).

### KIF11 Is an Independent Prognostic Factor

**Figure 5:**
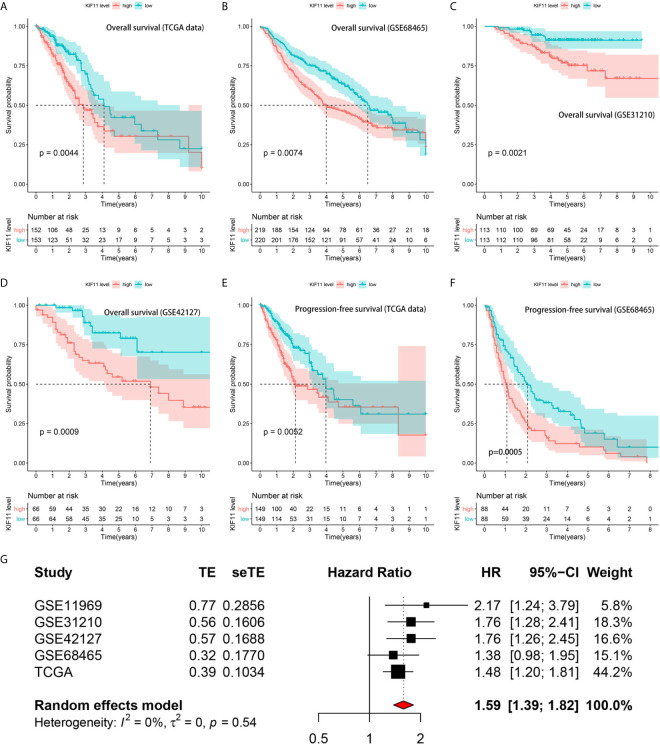
Correlation of *KIF11* expression with the LUAD patient survival. Higher *KIF11* expression predicted poor OS according to the **(A)** TCGA dataset, **(B)** GSE68465, **(C)** GSE31210, and **(D)** GSE42127 profiles. LUAD patients were classified into high and low *KIF11* expression subgroups relative to the median *KIF11* expression level. Higher *KIF11* expression predicted a worse PFS in both the **(E)** TCGA dataset and **(F)** GSE68465 profile. Meta-analysis associated *KIF11* expression with the OS in five cohorts **(G)**.

A Kaplan–Meier survival analysis indicated that high KIF11 expression is significantly associated with an unfavorable OS (**Figures 5A–D**) and poor PFS in LUAD patients (**Figures 5E, F**). Due to a lack of significant heterogeneity (*p >*0.05, I^2^ <50%), we selected a fixed model to perform the meta-analysis. The results showed that a high *KIF11* expression in LUAD patients indicated a lower OS (HR = 1.95 and 95%CI: 1.39–1.82, **Figure 5G**). A Cox regression analysis suggested that *KIF11* expression level is negatively correlated with the OS and PFS in LUAD (**Table 1**). Based on the above three methods, we used *KIF11* as an independent prognostic factor to predict cases of LUAD in our analysis. Furthermore, *KIF11* expression was significantly correlated with tumor stage of LUAD (**Supplementary Figures S3A, B**). The differences in *KIF11* expression between the T classification subgroups (T2–4 vs. T1), N classification subgroups (N1–3 vs. N0), M classification subgroups (M1 vs. M0), and gender (male vs. female) categories were also statistically significant, but that between age subgroups (>65 vs. <=65) was not (**Supplementary Figures S3C–G**). High *KIF11* expression was significantly correlated with an unsatisfactory OS in patients in stages I and II, T2–4, N0, M0, and female categories, but not in stages III and IV, T1, N1–3, M1, and male patient categories (**Supplementary Figures S3H–Q**). Additionally, *KIF11*expression was positively associated with the tumor grade (**Supplementary Figure S4**).

**Table 1:** Cox regression analysis for KIF11 expression on OS and PFS of LUAD patients.

Parameter	Univariate analysis			Multivariate analysis		
	HR	95%CI	*p*-value	HR	95%CI	*p*-value
**TCGA (OS)**						
Age	0.995	0.976–1.014	0.601	0.994	0.975–1.013	0.509
gender	0.873	0.592–1.288	0.494	0.859	0.579–1.274	0.451
stage	0.961	0.782–1.182	0.707	0.571	0.328–0.992	**0.047**
T classification	1.166	0.915–1.485	0.215	1.543	1.123–2.119	**0.007**
M classification	0.899	0.416–1.944	0.787	2.393	0.664–8.619	0.182
N classification	1.009	0.771–1.319	0.950	1.325	0.808–2.175	0.265
*KIF11*	1.475	1.205–1.807	**<0.001**	1.601	1.289–1.989	**<0.001**
**TCGA (PFS)**						
age	1.011	0.933–1.030	0.240	1.024	1.004–1.044	**0.017**
gender	1.251	0.859–1.822	0.243	1.016	0.692–1.492	0.935
stage	1.732	1.453–2.063	**<0.001**	1.674	1.384–2.024	**<0.001**
T classification	1.135	0.892–1.444	0.304	1.101	0.842–1.439	0.481
M classification	0.672	0.294–1.538	0.347	0.605	0.263–1.392	0.237
N classification	0.951	0.742–1.218	0.688	0.852	0.654–1.111	0.237
*KIF11*	1.443	1.183–1.761	**<0.001**	1.321	1.062–1.642	**0.012**
**GSE68465 (OS)**						
age	1.027	1.013–1.040	**<0.001**	1.031	1.017–1.045	**<0.001**
gender	1.436	1.107–1.863	**0.006**	1.218	0.933–1.588	0.147
grade	1.135	0.934–1.397	0.204	0.922	0.735–1.157	0.482
T classification	1.652	1.376–1.983	**<0.001**	1.417	1.171–1.715	**<0.001**
N classification	2.012	1.710–2.368	**<0.001**	2.033	1.724–2.396	**<0.001**
*KIF11*	1.001	1.000–1.002	**0.003**	1.001	1.000–1.003	**0.007**
**GSE68465 (PFS)**						
age	1.010	0.991–1.030	0.301	1.016	0.997–1.036	0.103
gender	1.225	0.871–1.722	0.243	1.144	0.806–1.623	0.451
grade	1.492	1.125–1.979	**0.006**	1.182	0.871–1.604	0.284
T classification	1.497	1.174–1.908	**0.001**	1.361	1.062–1.744	**0.015**
N classification	1.575	1.267–1.959	**<0.001**	1.541	1.231–1.928	**<0.001**
*KIF11*	1.002	1.001–1.003	**<0.001**	1.002	1.000–1.003	**0.008**

LUAD, lung adenocarcinoma; HR, hazard ratio; CI, confidence interval; OS, overall survival; PFS, progress-free survival. The bold values indicate the p-value less than 0.05.

### KIF11 Is Associated With Functions Underlying LUAD Progression

**Figure 6:**
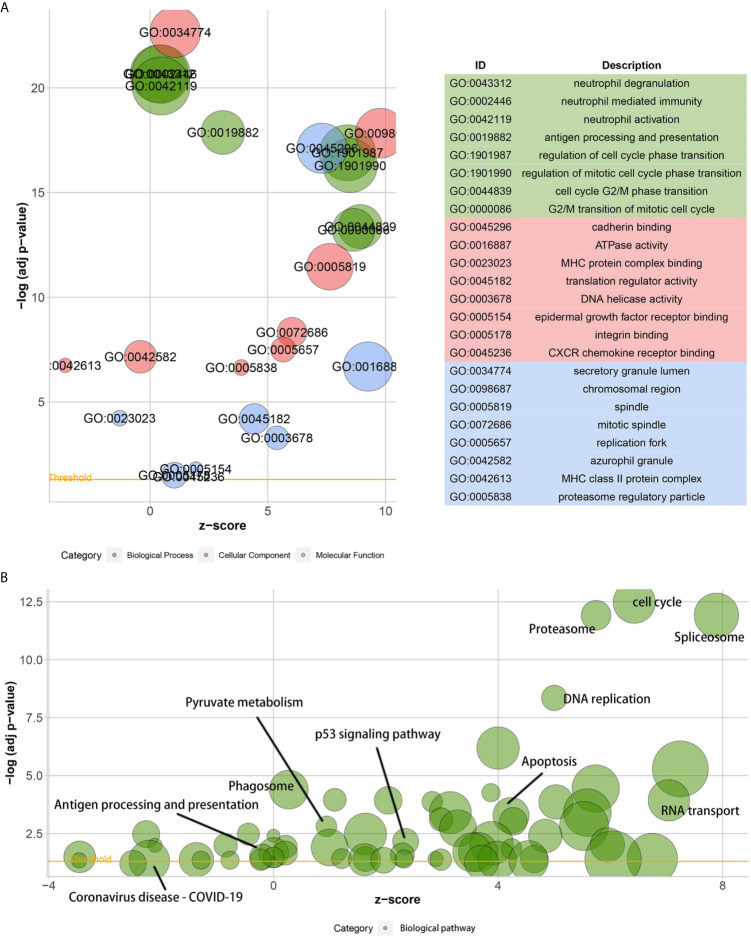
Gene ontology (GO) and the Kyoto encyclopedia of genes and genomes (KEGG) enrichment analyses. Bubble charts of the GO function analysis **(A)** and the KEGG pathway analysis **(B)** of DEGs among samples, which are divided into high and low *KIF11* expression subgroups relative to the median *KIF11* expression levels. The higher the z-score, the higher the expression of enriched terms.

A GO analysis suggested that the DEGs between low- and high-KIF11 expression subgroups were enriched in neutrophil activation immunity, regulation of cell cycle phase transitions, the cell cycle G2/M phase transition, and other biological processes (**Figure 6A**). ATPase activity, MHC protein complex binding, and DNA helicase activity were the primarily enriched terms of cellular components. The DEGs are involved in molecular functions including the chromosomal region, mitotic spindle, replication fork, and others (**Supplementary Table S3**). In addition, a KEGG analysis suggested that the DEGs were predominantly associated with cell cycle, spliceosome, proteasome, DNA replication, and other biological pathways (**Figure 6B**, **Supplementary Table S4**). Furthermore, GSEA results demonstrated that the cell cycle gene set had the highest enrichment scores in the KEGG collection (**Supplementary Figure S5** and **Table S5**). Gene sets enriched in biological processes and HALLMARK were also present.

### KIF11 Is Involved in the Formation of Tumor Microenvironment (TME)

**Figure 7:**
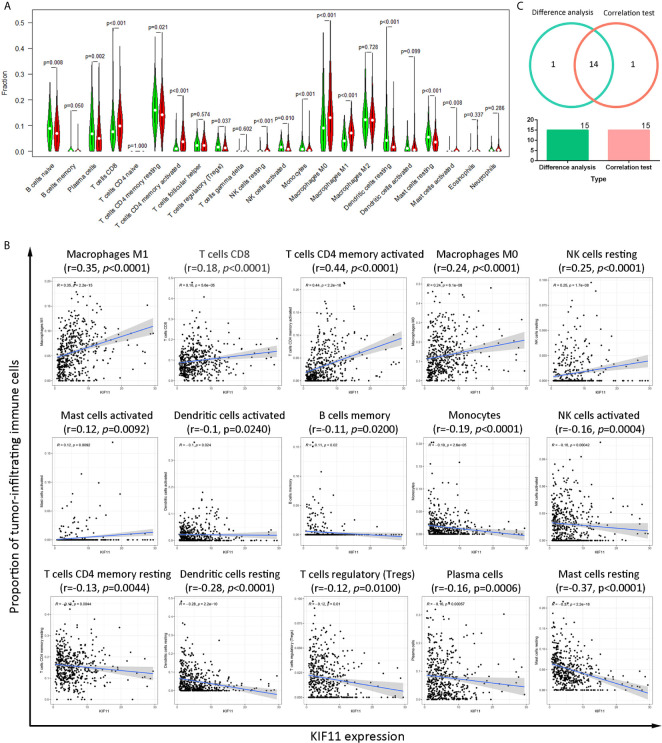
Correlation of the TICs proportions with *KIF11* expression. Analysis of differences in 22 TICs proportions between LUAD sample subgroups with high and low *KIF11* expression relative to the median *KIF11* expression level **(A)**. Correlation test of the proportions of 15 TICs with *KIF11* expression levels (p <0.05) **(B)**. Venn diagram shows 14 TICs that are correlated with *KIF11* expression levels as codetermined by the difference analysis and correlation test **(C)**.

A higher immune score predicted a favorable OS for LUAD patients, as well as favorable stromal and ESTIMATE scores (**Supplementary Figures S6A–C**). The immune score, stromal score, and ESTIMATE score were negatively associated with tumor stage (**Supplementary Figure S6D**), and there was statistical significance in the association between scores and *KIF11* expression (**Supplementary Figures S6E–H**). A total of 14 common TICs, codetermined by difference and correlation analyses, were associated with *KIF11* expression in LUAD samples (**Figure 7**). Additionally, resting NK cells and regulatory T cells were negatively correlated with OS, while resting memory CD4^+^T cells and monocytes were positively correlated with OS (**Supplementary Figure S7**).

### KIF11 Knockdown Inhibited Cell Proliferation and Induced Apoptosis

**Figure 8:**
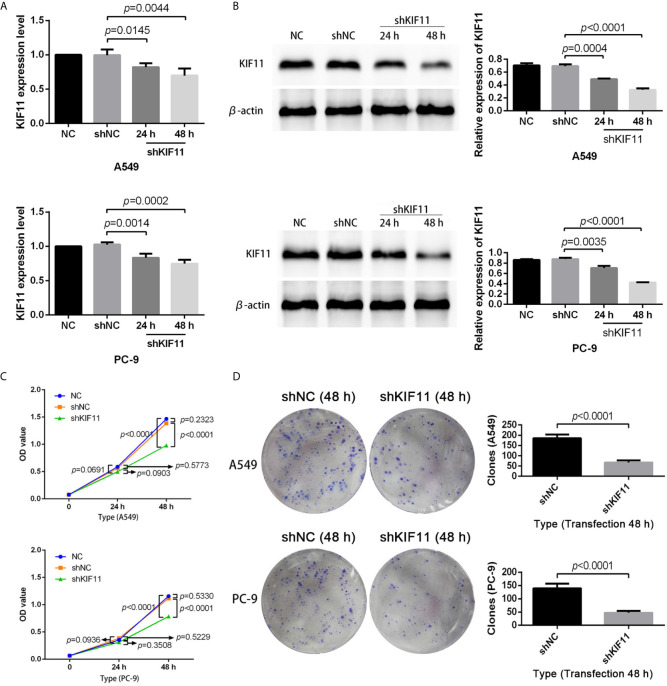
KIF11 knockdown inhibited the proliferation of LUAD cells. Efficiency of the KIF11 knockdown as determined by **(A)** quantitative real-time PCR and **(B)** Western blot analysis. Inhibitory effect of KIF11 knockdown on cell growth as measured by **(C)** CCK-8 test and **(D)** colony formation assay.

**Figure 9:**
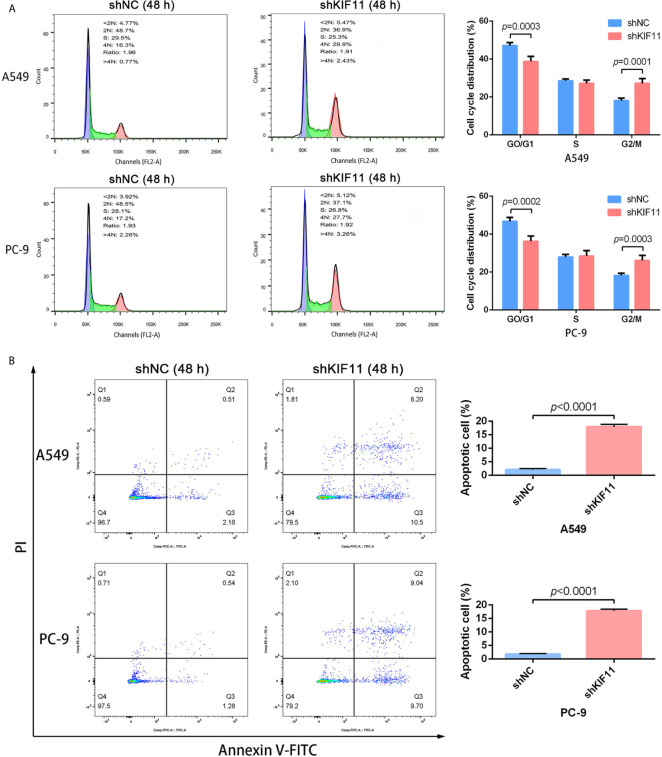
KIF11 knockdown induced the G2/M arrest and apoptosis of LUAD cells. KIF11 knockdown increased the proportions of A549 and PC-9 cells at the G2/M phase **(A)**. KIF11 knockdown promoted the percentages of apoptotic A549 and PC-9 cells **(B)**.

The results of qRT-PCR and Western blotting indicate that *KIF11* in A549 and PC-9 cells was efficiently knocked down (**Figures 8A, B**). A knockdown of *KIF11* (shKIF11) significantly reduced cell proliferation in A549 and PC-9 cells (**Figures 8C, D**) and resulted in a distinctive increase of the proportion of cells at the G2/M phase (**Figure 9A**). There was a remarkable increase in the percentage of apoptotic cells in the shKIF11 group versus the control (shNC) group (**Figure 9B**).

### KIF11 Knockdown Inhibited Cell Migration and Invasion

**Figure 10:**
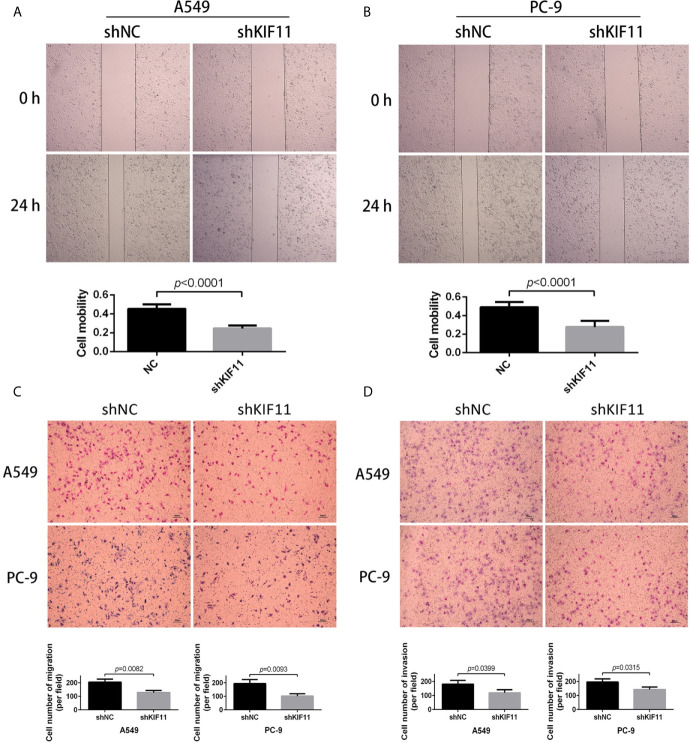
KIF11 knockdown inhibited the migration and invasion of LUAD cells. Wound healing assay revealing that KIF11 knockdown reduced the migration capability of **(A)** A549 and **(B)** PC-9 cells after 24 h transfection. Transwell assay showing that KIF11 knockdown decreased **(C)** migration and **(D)** invasion of A549 and PC-9 cells after 24 h transfection.

Furthermore, we found that a knockdown of *KIF11* exerted an inhibitory effect on cell migration in wound healing assays for A549 and PC-9 cells (**Figures 10A, B**), as observed in transwell migration analysis (**Figure 10C**). For invasion assays, the depletion of *KIF11*attenuated the invasive ability of A549 and PC-9 cells, as determined by the significant reduction of cell numbers in the lower chamber compared with the shNC group (**Figure 10D**).

## Discussion

LUAD is a malignant cancer with high morbidity and mortality (1, 2). Despite the basic approaches of surgery, radiotherapy, and chemotherapy that have contributed to the improved clinical prognosis and survival of tumor patients, LUAD is still challenging to treat due to a poor understanding of the molecular mechanisms and basic signaling pathways in physiological processes of lung cancer. Molecule-targeted therapy is expected to be a novel treatment strategy for solid tumors, but its efficacies and benefits remained limited (22). Therefore, the development of a novel and efficient molecular target for LUAD treatment is necessary. In this work, *KIF11* was identified as a hub gene with an integrated bioinformatics analysis and validated in extended experiments. KIF11 is a kinesin that is primarily responsible for intracellular vesicle transport and mitosis in addition to being overexpressed in various tumors (23–25). High *KIF11* expression significantly predicted an unacceptable overall and progression-free survival and was correlated with advanced tumor stage and grade. Of note, the gene was also identified as a prognostic factor *via* a meta-analysis and Cox regression analysis. Further studies demonstrated that a knockdown of *KIF11* had inhibitory effects against cell proliferation, migration, and invasion, in addition to inducing cell cycle arrest and apoptosis in LUAD cells. These data imply that *KIF11* may be a promising therapeutic target for LUAD.

It has been reported that KIF11 plays essential roles in G2/M phase transition and cell cycle checkpoints during mitosis, subsequently modulating tumor progression (26, 27). Jiang et al. found that high *KIF11* expression was correlated with triple negative breast cancer (TNBC) and indicated poor disease-free survival (28). *KIF11* silencing with a KIF11 inhibitor suppressed cell growth and induced apoptosis in TNBC cells in TNBC xenograft models. Zhou et al. also demonstrated that suppressing *KIF11* expression disrupted cell growth, migration, and invasion, but promoted apoptosis in breast cancer (23), which is consistent with our observations in LUAD tissues. Furthermore, KIF11 knockdown significantly reduced tumor size and weight, which might be due to downregulation of N-cadherin and vimentin as well as reductions in ERK, AMPK, AKT, and CREB phosphorylation. SB743921, a specific KIF11 inhibitor, significantly suppressed cell proliferation, migration, and epithelial to mesenchymal transition process, in addition to inducing apoptosis in clear cell renal cell carcinoma, which together indicate the dominant roles of KIF11 in tumor pathogenesis (29). Additionally, *KIF11*suppression may strengthen the cytotoxicity of adriamycin in breast cancer cell lines (MCF-7 and MDA-MB-231) (30). This effect was validated in an extended population study, which also suggested that low expression of *KIF11* in early-stage breast cancer patients was significantly associated with prolonged survival time after chemo-and radiotherapy. Regarding LUAD, there were only two reports that mentioned *KIF11* as a part of a gene signature (31, 32), however, the prognostic value and functions of *KIF11* were not clearly elaborated.

On the other hand, *KIF11* is also associated with cell mobility. Relative to mass spectrometric analysis, Shi et al. have found that KIF11 was co-purified with death receptor 6 (DR6), which could promote cellular migration capacity mediated by MAPK/ERK and PI3K/AKT signaling pathways for ovarian carcinoma (33). Meanwhile, *KIF11* could reduce the inhibitory effects of DR6 knockdown on ovarian carcinoma cell migration, implying *KIF11*, to some extent, contributed to the cell mobility. Besides, KIF11 could act as a microtubule motor and was a component of β-actin messenger ribonucleoprotein particles (mRNPs). It was demonstrated that KIF11 interacted with ZBP1, an mRNA-binding protein, to manipulate the mRNPs transport, mediate cell polarity, promote cellular structure asymmetry, and subsequently regulate cell migration (34). A previous study reported that dimethylenastron, as a specific inhibitor of KIF11, significantly suppressed the migratory and invasive ability in PANC1 pancreatic cancer cells, but not their proliferative potential (35). Further research has found that dimethylenastron could inhibit the motor domain ATPase of KIF11. All the results indicated that *KIF11* has potential to regulate the cell mobility. However, there was no study for the functions of *KIF11* on cell mobility in LUAD.

It is well known that the TME is widely involved in tumor progression and primarily contains malignant and nonmalignant cells (36, 37). Malignant cells either interact with surrounding components to enhance their proliferation and metastasis capabilities or spread to other healthy tissues to take part in the initiation and progression of solid tumors (38–40). Nonmalignant cells in the TME are thought to have beneficial effects on carcinogenesis by improving the proliferative abilities of potentially malignant cells (41–43). Previous studies have suggested that the reciprocal interactions between malignant cells in the TME may result in the recruitment, activation, and reprogramming of immune and stromal cells, as well as modulation of cancer progression (44, 45). A growing number of works have focused on the importance of the immune microenvironment in tumorigenesis (46–48). Our results suggest that four TICs (resting NK cells, resting memory CD4^+^ T cells, regulatory T cells, and monocytes) were significantly correlated with *KIF11* expression, and were highly correlated with the OS in LUAD patients.

Natural killer (NK) cells are a part of the innate immune system that both mediate cellular cytotoxicity without prior activation and play a critical role in cancer immune surveillance (49, 50). A previous study reported that NK cells with high cytotoxicity were positively correlated with a longer OS in patients with metastatic prostate cancer (mPC) (51). However, immunosuppressive cytokines or other soluble factors in the TME, such as soluble NKG2D ligand and tumor growth factor-β, impaired NK cell cytotoxicity by targeting the activating receptor NKG2D,inhibiting its interaction with membrane-bound ligands on tumor cells (52, 53). CD4^+^ T cells are another major cell community that controls tumor growth. Li et al. found that fractions of peripheral CD4^+^ T cells were positively correlated with tumor size in gastric cancer patients (54). Conversely, a high density of infiltrating CD4^+^ T cells indicates improved relapse-free survival and disease-specific survival in colorectal cancer patients (55). Among CD4^+^ T cells, central memory cells are primarily responsible for immune memory and immune protection during tumor metastasis while effector memory cells play essential roles in regulating the expression of adhesion molecules and chemokine receptors (56–58). CD4^+^ regulatory T (Treg) cells were demonstrated to have important roles in the maintenance of self-tolerance and immune homeostasis (59, 60). Treg cells infiltrate multiple tumor tissues and often serve as inhibitors of antitumor immunity. Reducing Treg cell infiltration is reported to rescue antitumor immunity in animal models (61, 62). Higher proportions of Treg cells among TICs, especially the elevated ratio of Treg to CD8^+^ T cells, often indicate unfavorable survival or prognosis (59, 63). Monocytes are a large portion of innate immune cells, serving as an important regulator of tumorigenesis and enlargement (64, 65). The signals range from being immunosuppressive to being immunostimulatory, which make monocyte subsets differentially responsive to the surrounding microenvironment and even display opposite functions (64, 66). In malignant tumors, infiltrating monocytes initially perform antitumor functions by preventing tumor metastasis (67, 68). Overtime, some functional (e.g., M-CSF and GM-CSF) and transcriptional (e.g., IRF4 and MAFB) factors in the TME induce monocyte differentiation into pro-tumoral, tumor-associated macrophages and dendritic cells, which help tumor cells avoid cytotoxic T cells (69–71). Therefore, clearly understanding the correlation between immune cells and tumor cells could contribute to the development of a novel and efficient therapy strategy for tumorigenesis.

This work contributes to the understanding of potential molecular mechanisms of LUAD pathogenesis but has some limitations. First, the functions of *KIF11* were verified *in vitro* but not *in vivo*, which will be addressed in future studies. Second, despite the effects of KIF11 on cell cycle and in inducing apoptosis in LUAD cells, the molecular mechanisms behind these observations are still not clear. Third, correlations of *KIF11* with TICs were elucidated based on bioinformatics analysis, but not experimentally validated. Finally, *KIF11* was identified as a hub gene based on a TCGA dataset with limited samples and unbalanced clinical data, thus, the efficacy of *KIF11* as a therapeutic target and prognostic factor needs further validation. In summary, the work demonstrates that *KIF11* is overexpressed in LUAD tissues. High *KIF11* expression significantly predicts poor OS and PFS in LUAD patients, and *KIF11* was further implicated in alteration of the TME and TIC infiltration. *KIF11* knockdown inhibited cell proliferation by inducing the G2/M phase arrest and promoting apoptosis in A549 and PC-9 cells. In addition to suppressing growth, depletion of *KIF11* reduced the migratory and invasive capabilities of A549 and PC-9 cells. These findings indicate that *KIF11* may be an independent prognostic factor and promising therapeutic target for LUAD patients.

## Data Availability Statement

The datasets presented in this study can be found in online repositories. The names of the repository/repositories and accession number(s) can be found in the article/supplementary material.

## Author Contributions

ZL and FL designed the study. ZL, BY, and FQ performed the experiments. ZL, BY, and FL analyzed the data. BY, FQ, and FL prepared the figures. ZL and FQ wrote the manuscript. BY and FL supervised the study. All authors contributed to the article and approved the submitted version.

## Conflict of Interest

The authors declare that the research was conducted in the absence of any commercial or financial relationships that could be construed as a potential conflict of interest.
