# Supplementary material for: KIF11 Serves as an Independent Prognostic Factor and Therapeutic Target for Patients With Lung Adenocarcinoma
Source: Front Oncol. 2021 Apr 23;11:670218. doi: 10.3389/fonc.2021.670218 (PMC8103954; doi:10.3389/fonc.2021.670218)
Supplement: Supplementary file 1 [file Table_1.docx]

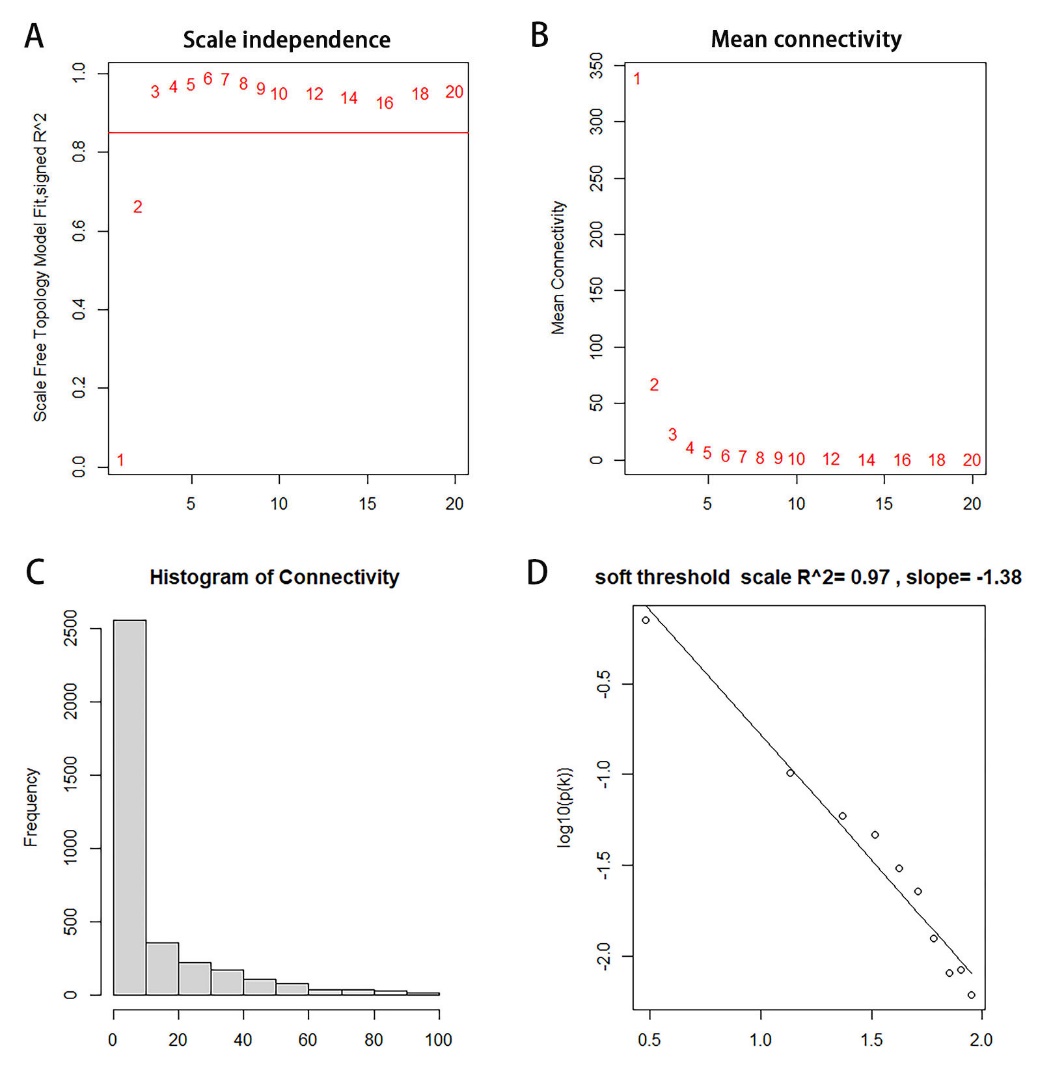


**Figure S1.** Determination of the soft‑threshold power in the weighted gene co-expression network analysis (WGCNA). Analysis of the (A) scale‑free fit index and the (B) mean connectivity for various soft-thresholding powers (β). Histogram of the (C) connectivity distribution and (D) validation of the scale-free topology when β=5.


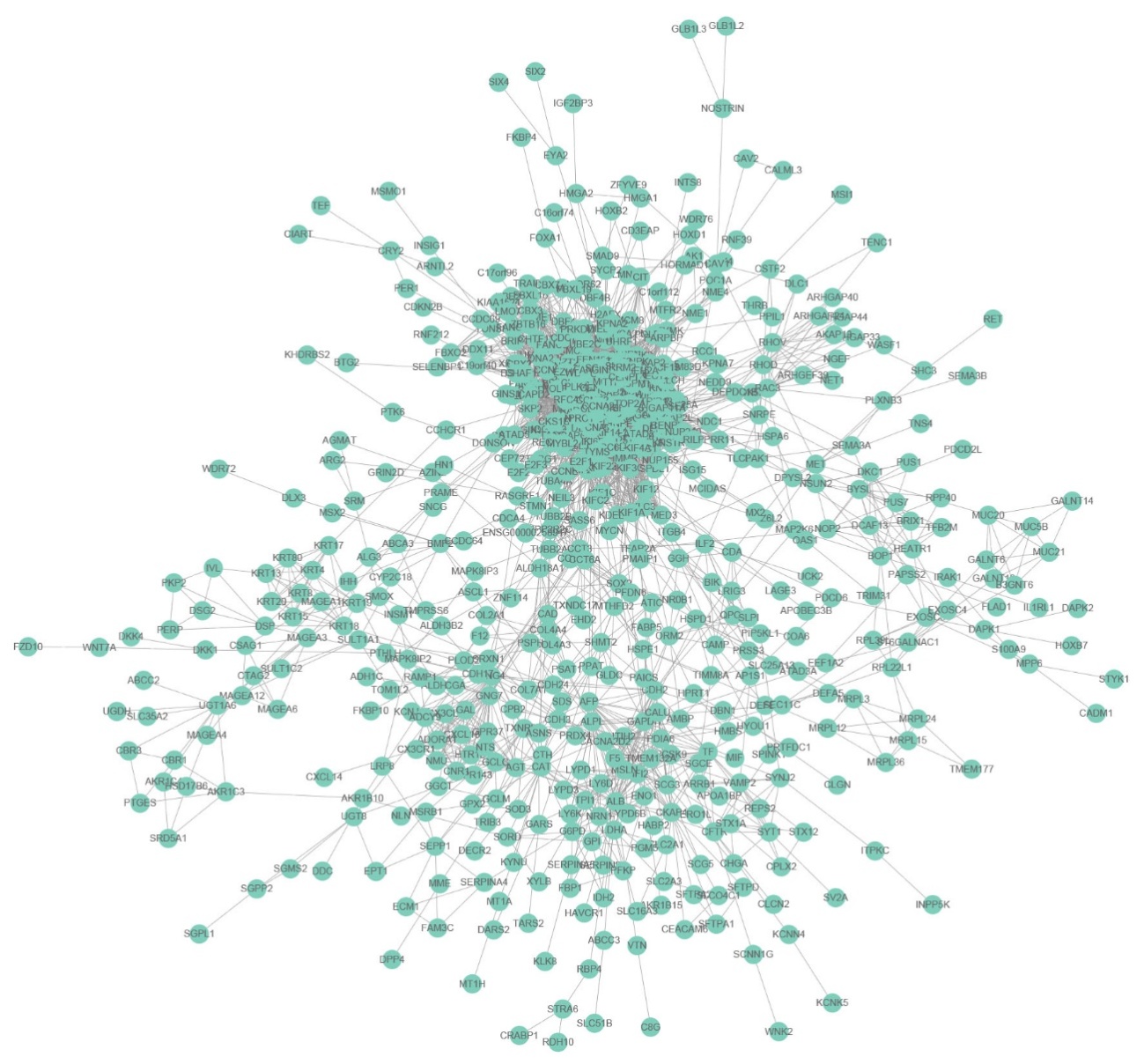


**Figure S2.** Construction of the PPI network based on the differentially expressed genes in the turquoise module.


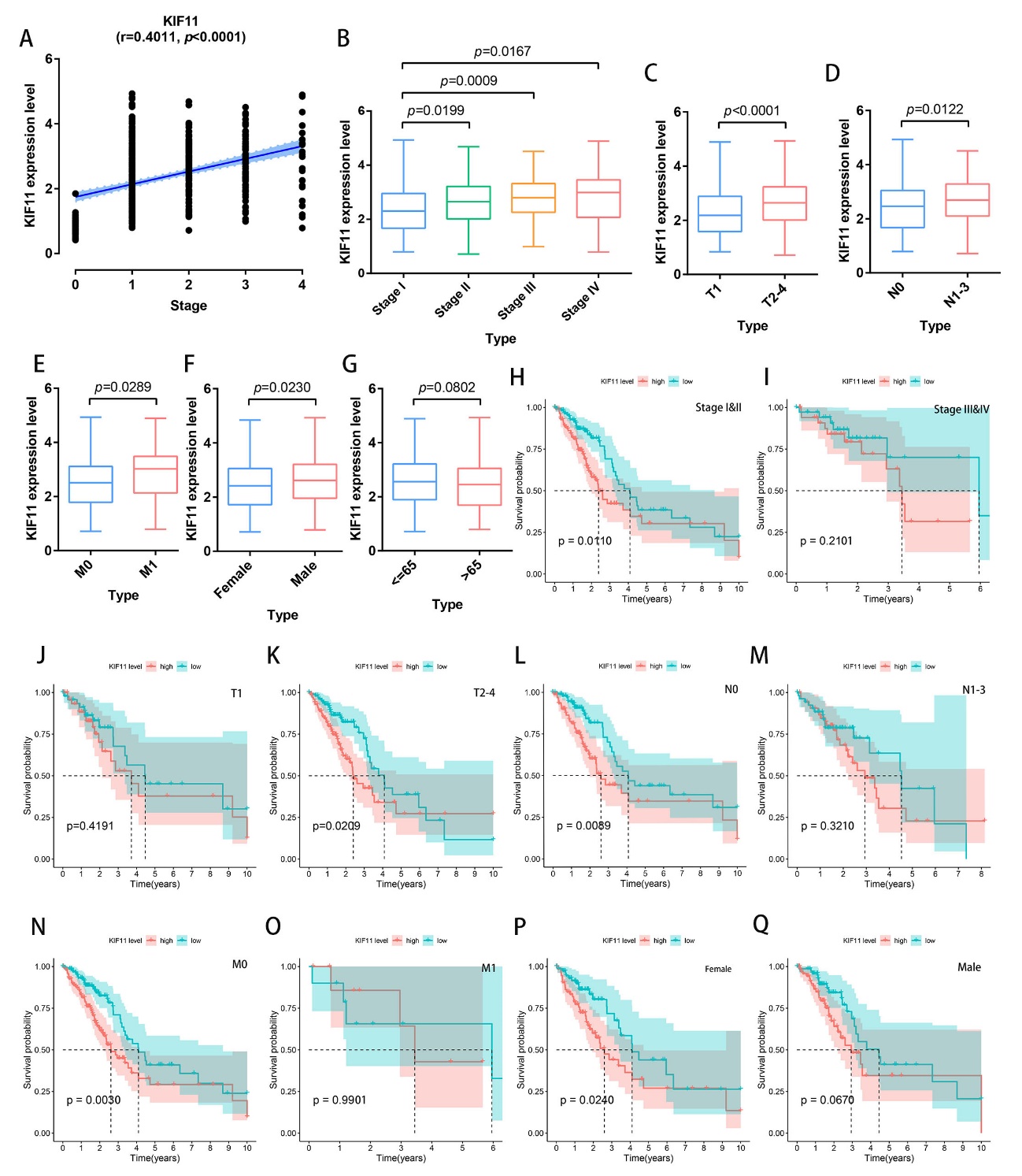


**Figure S3.** The prognostic significance of *KIF11* expression based on the TCGA dataset. *KIF11* expression is significantly correlated with tumor stage (A). *KIF11* expression distribution across tumor stages (B). *KIF11* is highly expressed in (C) T2-4 vs. T1, (D) N1-3 vs. N0, (E) M1 vs. M0, and (F) male vs. female subgroups, but not between (G) age subgroups. Correlation of *KIF11* expression with OS in LUAD patients with clinical characteristics that include the tumor stage: (H) stages I and II, (I) stages III and IV, primary tumors: (J) T1 and (K) T2-4, lymph node metastasis: (L) N0 and (M) N1-3, distant metastasis: (N) M0 and (O) M1, and gender: (P) female and (Q) male.


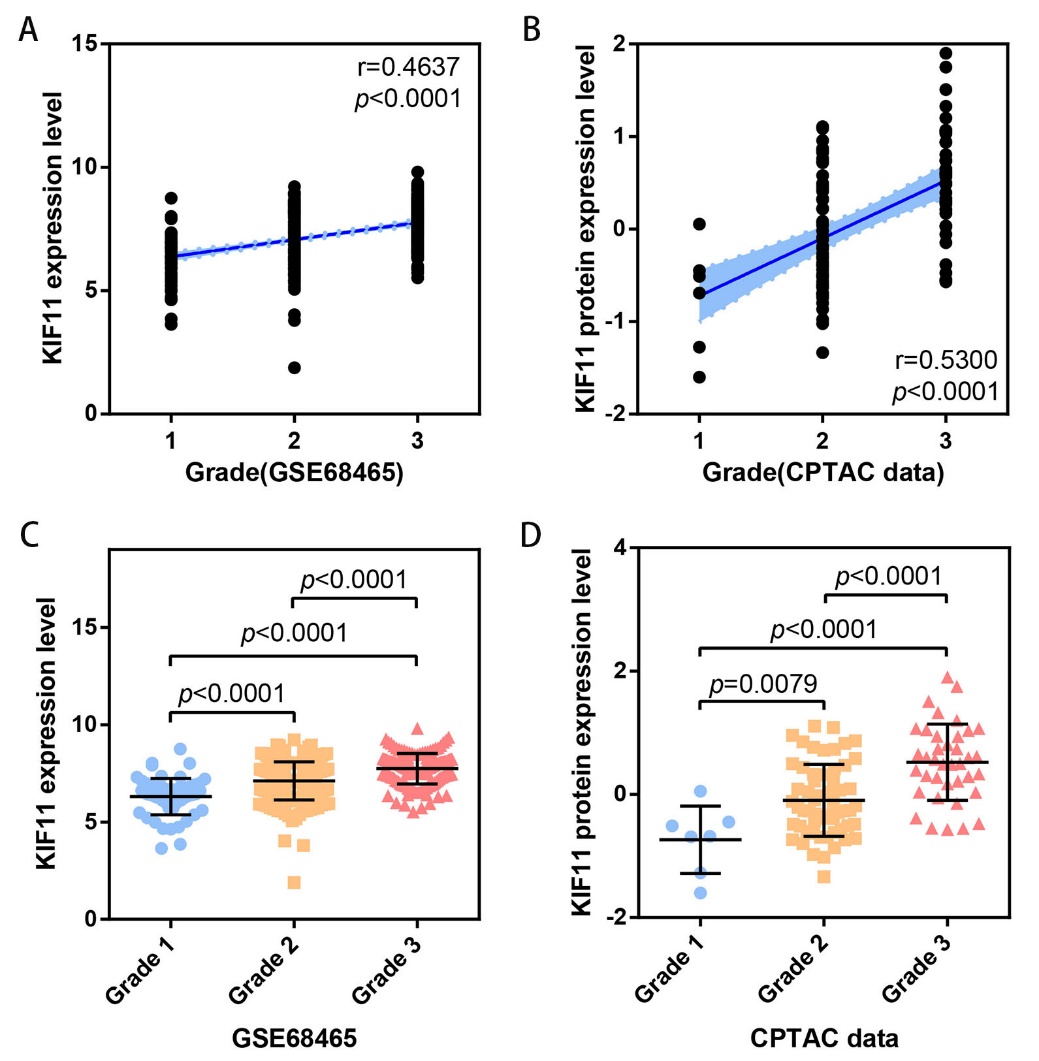


**Figure S4.** *KIF11* expression is positively associated with the tumor grade in LUAD patients. Correlation analyses of *KIF11* expression with the tumor grade based on (A) the GSE68465 profile and (B) CPTAC data. Difference analyses of the *KIF11* expression level in LUAD patients with differing clinical grades according to (C) the GSE68465 profile and (D) CPTAC data.


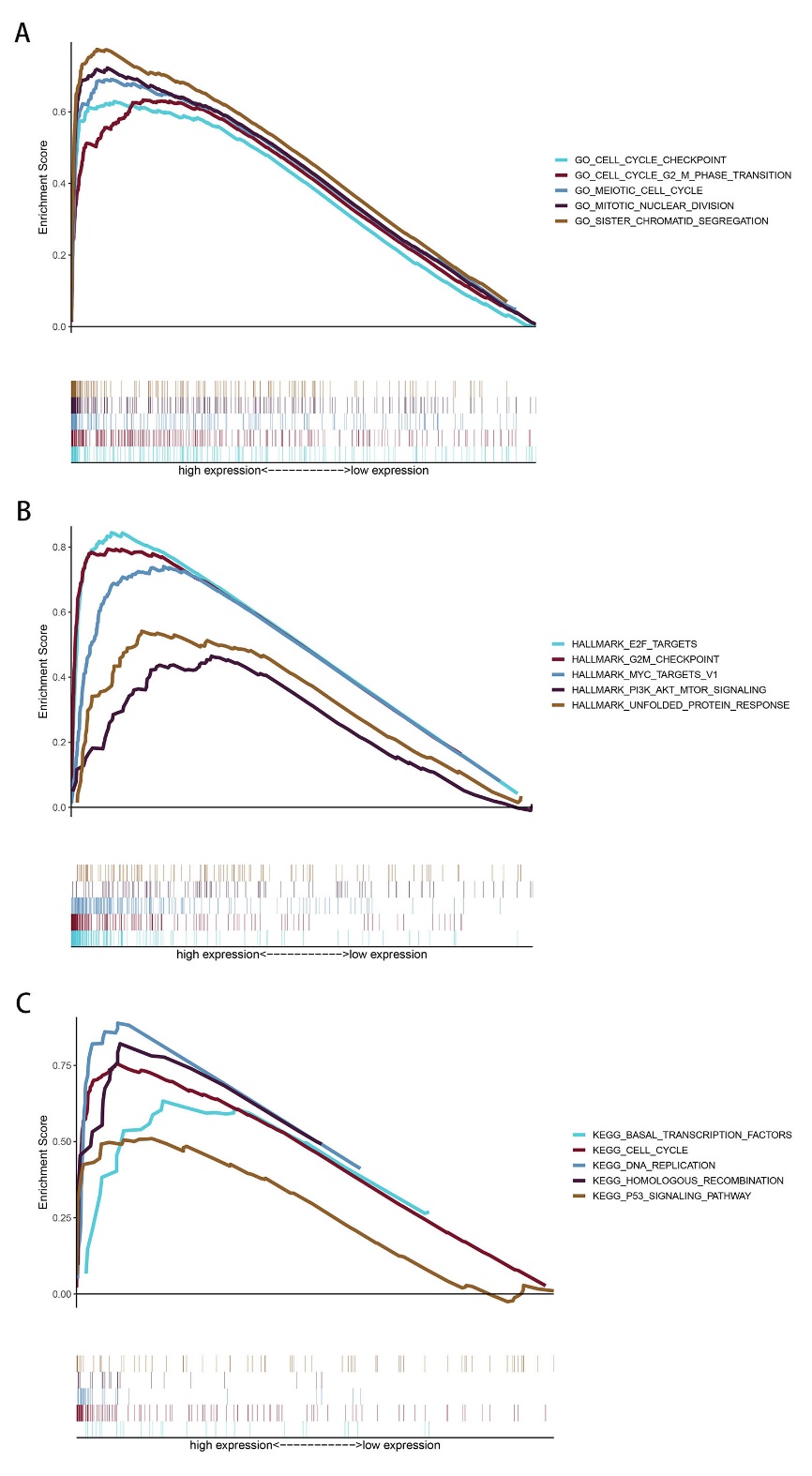


**Figure S5.** Gene set enrichment analysis (GSEA) of LUAD samples with high *KIF11* expression. The gene sets enriched in the biological process of gene ontology (GO) from high *KIF11* expression level samples (A). Only five typical gene sets are displayed. The high or low expression of *KIF11* was determined by the median *KIF11* expression level. The enriched gene sets in the HALLMARK collection from the high *KIF11* expression samples (B). The enriched gene sets in the Kyoto encyclopedia of genes and genomes (KEGG) collection from the high *KIF11* expression level group (C).

**
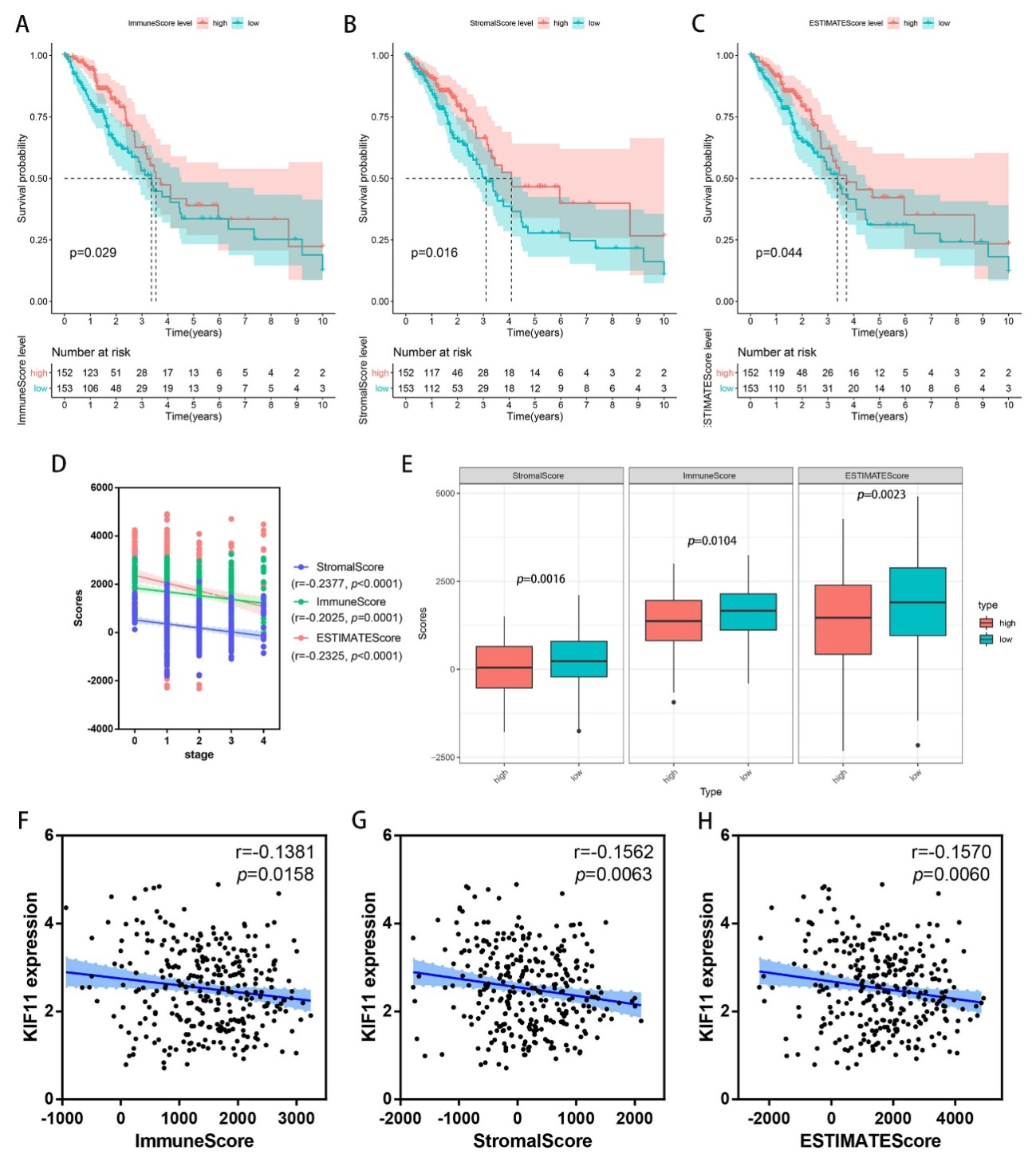
**

**Figure S6.** Correlation of tumor microenvironment (TME) scores with OS and *KIF11* expression. Kaplan-Meier survival analysis showing the association of the (A) immune score, (B) stromal score, and (C) ESTIMATE score with the OS of LUAD patients with low and high TME scores, based on the corresponding score medians. Analysis of the correlation between scores and tumor stage (D). Analysis of differences in scores between LUAD sample subgroups with high and low KIF11 expression relative to the median *KIF11* expression level (E). Analysis of the correlations between (F) immune score, (G) stromal score, and (H) ESTIMATE score and the *KIF11* expression level.


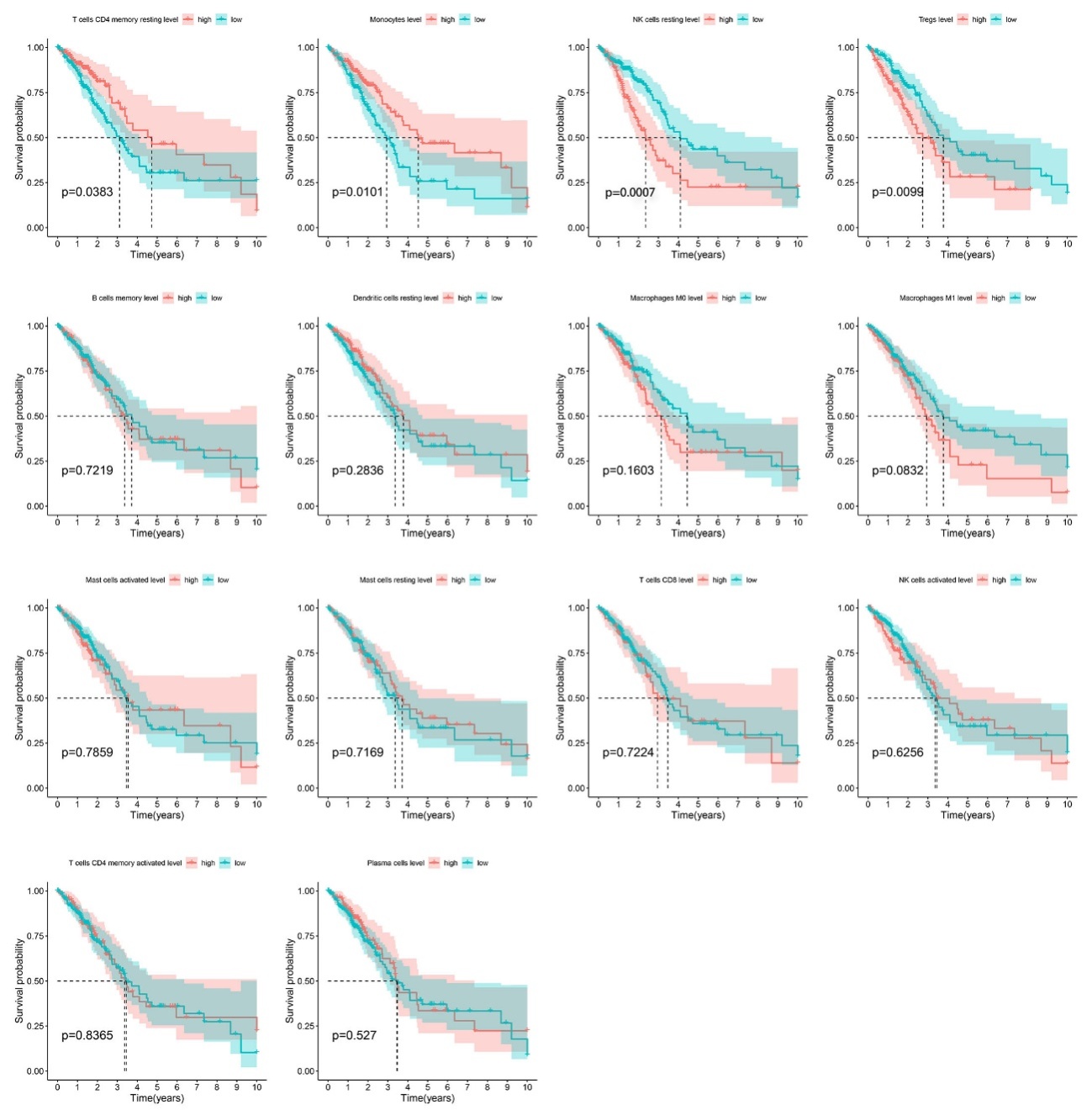


**Figure S7.** Correlation of tumor-infiltrating immune cells (TICs) proportions with the OS in LUAD patients. A Kaplan-Meier survival analysis shows the association of 14 TICs with OS in LUAD samples, which were grouped into low and high TIC subgroups according to the corresponding TIC median proportion.
